# Intranasal 1-Methyl-4-phenyl-1,2,3,6-tetrahydropyridine (MPTP) Administration Hampered Contractile Response of Dopamine in Isolated Rat Ileum

**DOI:** 10.3390/biomedicines13102400

**Published:** 2025-09-30

**Authors:** Ana Silva, Sofia Viana, Inês Pita, Cristina Lemos, Filipe C. Matheus, Lina Carvalho, Carlos A. Fontes Ribeiro, Rui D. Prediger, Frederico C. Pereira, Sónia Silva

**Affiliations:** 1 Institute of Pharmacology and Experimental Therapeutics, Faculty of Medicine, University of Coimbra, 3000-548 Coimbra, Portugal; ana.silva.1@gmail.com (A.S.); sofia_viana@estesc.ipc.pt (S.V.); inespita1989@gmail.com (I.P.); fontes.ribeiro@gmail.com (C.A.F.R.); 2 Coimbra Institute for Clinical and Biomedical Research (iCBR), Faculty of Medicine, University of Coimbra, 3000-548 Coimbra, Portugal; 3 Centre for Innovative Biomedicine and Biotechnology (CIBB), University of Coimbra, 3004-504 Coimbra, Portugal; 4 Coimbra Health School, Health and Technology Research Center (H&TRC), Polytechnic University of Coimbra, 3046-854 Coimbra, Portugal; 5 Department of Psychiatry, University of Innsbruck, 6020 Innsbruck, Austria; cristina.lemos@curie.fr; 6 Department of Pharmaceutical Sciences, Federal University of Santa Catarina (UFSC), Florianópolis 88040-900, Brazil; filipejfmatheus@gmail.com; 7 Institute of Anatomical and Molecular Pathology, University of Coimbra, 3004-504 Coimbra, Portugal; linacarvalho58@gmail.com; 8 Department of Pharmacology, Center of Biological Sciences, Federal University of Santa Catarina (UFSC), Florianópolis 88040-900, Brazil; rui.prediger@ufsc.br; 9 Faculty of Pharmacy, University of Coimbra, 3000-548 Coimbra, Portugal

**Keywords:** Parkinson’s disease, intranasal MPTP, GI dysfunction, dopamine, D2-like receptors, enteroglial cells

## Abstract

**Background**: Gastrointestinal (GI) disturbances occur frequently in the early premotor stage of Parkinson’s disease (PD). These GI impairments are associated, at least in part, with dopaminergic dysfunction in the myenteric plexus. However, the enteric nervous system (ENS) pathophysiology underlying GI dysfunction in PD has been overlooked. **Objectives**: The aim of this study was to evaluate the premotor GI disturbances in rats submitted to intranasal (i.n.) MPTP, a valid experimental model of the premotor stage of PD. **Methods**: Ileum segments from male Wistar rats (21 weeks old) were collected 12 days following the i.n. MPTP administration for functional studies. Isometric contractile concentration–response (CR) curves (cumulative) for dopamine (DA) were performed in both the presence and absence of sulpiride, a selective dopamine D2-like receptor (D2R) antagonist. **Results**: Functional studies showed that DA induced a concentration-dependent contractile response in the ileum, which exhibited marked contraction at lower concentrations (0.01–0.9 µM) and relaxation at higher concentrations (3–90 µM). MPTP significantly attenuated both the contraction and the ensuing relaxation. Furthermore, sulpiride significantly reduced the contractile response to DA in the control group and blocked the relaxation in the MPTP group. The MPTP-induced dysmotility occurred with preserved DA homeostasis, as shown by normal DA, TH, and D2R ileal levels in the MPTP group. However, MPTP seemed to impose a decrease in S100β and GFAP (enteroglial markers) immunostaining in the ileal myenteric plexus. **Conclusions**: In summary, we provide pioneering functional, neurochemical, and morphological evidence showing that rats submitted to the i.n. MPTP model exhibited premotor DA-dependent ileum motile dysfunction accompanied by enteroglial disturbance within the myenteric plexus, but with preserved DA markers.

## 1. Introduction

Parkinson’s disease (PD) is neuropathologically characterized by a progressive loss of dopaminergic neurons within the substantia nigra (SN), mainly affecting the ventral region of the pars compacta, which translates into increasingly debilitating motor function impairment [1,2].

However, several non-motor manifestations, including disturbances of sleep, loss of smell, depression, gastrointestinal (GI) disturbances, hypotension, and sweating, may occur and precede the onset of motor symptoms by more than a decade [2,3,4,5,6,7]. GI disturbances, such as gastric emptying impairments and constipation, are prominent amongst these premotor symptoms [8,9,10,11]. Dilatation of the small intestine and abnormalities in small intestine motor patterns have also been reported in PD [12,13]. Importantly, recognition and evaluation of GI dysfunction symptoms in PD [13,14,15,16] may lead to earlier and potentially more effective diagnostic and therapeutic intervention.

Central nervous system (CNS) nigral cells and many enteric nervous system (ENS) neurons share the capability of producing dopamine (DA), which regulates normal gut motility [12,17]. It was suggested that the D2-like receptor (D2R) is a major mediator of the effects of endogenous DA in the ENS [18,19]. It has been reported that PD patients present lower DA levels in the GI [20], suggesting that damage to the enteric dopaminergic (DAergic) system might be an important factor underlying GI dysfunction in PD. However, other authors using postmortem tissues and colon biopsies did not report DAergic enteric neuronal loss [21,22,23,24].

There is mounting evidence supporting the role of astrocytes in the inflammatory processes in PD [25,26]. Indeed, S100β (an astrocytic protein) is upregulated in the brains of PD patients [27]. Additionally, it was demonstrated that brain areas that are affected early in the development of PD have particularly high levels of glial fibrillary acidic protein (GFAP) and that there is an enteric glial reaction that leads to the overexpression of GFAP in PD patients [28,29,30]. However, only a few studies have investigated the inflammatory response, including glial reactivity, in the guts of PD patients [31,32].

In recent years, the ENS has been recognized as a player in the development of neurodegenerative diseases [33]. Excitingly, PD can be divided into two distinct subtypes based on the initial site of α-synuclein pathology and its spreading pattern [34]. In the brain-first subtype, α-synuclein pathology initially starts in the brain and subsequently spreads to the peripheral autonomic nervous system. Conversely, in the body-first subtype, the pathology arises in the enteric or peripheral autonomic nervous system and then spreads to the brain. The body-first subtype gives credence to the possibility that the ENS is a gateway for PD pathology, and environmental xenobiotics can trigger PD [35,36].

Indeed, the proneurotoxin 1-methyl-4-phenyl-1,2,3,6-tetrahydropyridine (MPTP) is known to cause a severe and irreversible PD-like syndrome both in humans and non-human primates [37]. A single intranasal (i.n.) administration of MPTP in rodents inflicts progressive non-motor and motor symptoms that closely recapitulate different PD stages. These symptoms, which include impairments in olfactory, cognitive, emotional, and motor functions, are associated with time-dependent disruption of DAergic neurotransmission (for review, see [38,39]). Moreover, it was postulated that after nasal administration, a part of the dose is swallowed and, thereafter, reaches the gut [40]. This might contribute to putative gut toxicity in the i.n. MPTP model. Thus, this experimental model, in addition to mimicking the temporal course of the appearance of different PD symptoms, also mimics the theory of olfactory and gastrointestinal vectorization so that environmental contaminants may be involved in the etiology of PD [41].

Therefore, in the current study, the ileal contractility and the underlying DAergic and enteroglial status in rats submitted to i.n. administration of MPTP, before the development of motor impairments, was evaluated to better characterize GI disturbances in this model of PD.

## 2. Materials and Methods

### 2.1. Animals and MPTP Administration Protocol

Male Wistar rats (14 weeks old; Charles River Laboratories) were housed two per cage in standard cages (49 cm in length; 34 cm in width; 16 cm in depth) and maintained under controlled environmental conditions (12 h light/dark cycle at 23 ± 1 °C and 60 ± 5% humidity) with food and water supplied ad libitum. The experimental protocol was approved by our Institutional Animal Care and Use Committee (ORBEA 55-2014/0406), and the procedures, performed by licensed users by the Federation of Laboratory Animal Science Associations (FELASA), conformed to the guidelines from Directive 2010/63/EU of the European Parliament for the Protection of Animals Used for Scientific Purposes. The animals were randomly allocated to two groups: control (*n* = 6) and MPTP (*n* = 6).

MPTP HCl (Sigma-Aldrich, St. Louis, MO, USA) was administered by the intranasal route (i.n.) according to the procedure described by Prediger et al. [41]. The rats were lightly anesthetized with 0.96% isoflurane (0.75 CAM; Abbott, Maidenhead, UK), delivered via a vaporizer system (SurgiVet Inc., Waukesha, WI, USA). A 10 mm segment of PE-50 tubing was gently inserted into one nostril and connected to a peristaltic pump operating at 12.5 μL/min. MPTP HCl was prepared in 0.9% saline at 20 mg/mL and infused for 4 min, corresponding to 1 mg per nostril. After a brief 1-min pause to allow recovery of normal respiration, the same infusion procedure was carried out through the opposite nostril. All handling of MPTP followed established safety guidelines. Animals in the control group were given saline.

### 2.2. Tissue Collection and Processing

Animals were sacrificed 12 days after MPTP administration by decapitation under isoflurane anesthesia, and the striata and the ilea (the last 10 cm before the ileocecal transition) were rapidly collected to perform the following analyses: (1) functional studies—fresh tissues (ileum); (2) quantification of DA levels by high-performance liquid chromatography (HPLC)—frozen tissues (striatum and ileum); (3) measurement of tyrosine hydroxylase (TH), D2R, and GFAP densities by Western blotting (WB)—frozen tissues (ileum and striatum); and (4) analysis of D2R cellular localization and of S100β and GFAP densities by immunohistochemistry (IHC)—fixed tissues (ileum). We used the ileum, herein, because this gastrointestinal segment is involved in nutrient digestion and absorption. Moreover, the ileum is considered a standard model for studying the pharmacological action of compounds within the intestine [42,43].

### 2.3. Functional Studies

The ilea were cut into 15 mm segments, excess fat and connective tissue were trimmed off, and the lumen was rinsed with a Krebs–Henseleit solution (mmol/L: NaCl, 118.67; KCl, 5.36; MgSO_4_·7H_2_O, 0.57; CaCl_2_·2H_2_O, 1.90; KH_2_PO_4_, 0.90; NaHCO_3_, 25.0; glucose, 11.1; pH 7.4). Then, the ileal segments were suspended on stainless-steel hooks according to the longitudinal smooth muscle layer orientation under a passive force of 29.6 mN in 15 mL organ baths filled with the physiological solution aerated with 5% CO_2_–95% O_2_ and maintained at 37 °C [44]. The ileal segments were stretched stepwise to their optimal point of tension–length relationship and allowed to equilibrate for a period of 2 h with repetitive changes of the buffer, having reached a basal tension of no less than 14.7 mN. The tension was increased or decreased as necessary until a stable baseline tension was obtained. This procedure allowed us to obtain quiescent tissues with little or no spontaneous activity. Tonic isometric contraction recordings with a Letica Scientific Instruments isometric transducer connected to a four-channel polygraph (Polygraph 4006; Letica Scientific Instruments, Panlab, Barcelona, Spain) ensued. Following the equilibration period, all intestinal segments were submitted to 100 µM of exogenous acetylcholine (ACh) (Sigma-Aldrich) to characterize the maximum contraction of ileum smooth muscle. Cumulative concentration–response (CR) curves for DA (0.01–90 µM) (Sigma-Aldrich) were then performed either in the absence or in the presence of 10 µM of sulpiride (Sigma-Aldrich), a selective D2R antagonist [45], added to the organ bath 30 min before the onset of the DA CR curve. Cumulative CR curves for DA were obtained because the recorded tonic isometric contractions displayed a stable plateau, which enabled the sequential addition of doses. The ileal segments were challenged again with ACh at the end of each experiment to confirm the sustained functional viability of the organ over the course of time. Ileal segments that failed to produce stable contractile responses to ACh throughout the experiment were not included in the final data. Contractile responses to DA were normalized to the maximal response of each preparation to exogenous ACh; thus, the data are expressed as percentages of ACh-induced maximum contraction. When using sulpiride, control segments were used with the appropriate solvent.

### 2.4. HPLC Analysis of DA Content

DAergic status was assessed by measuring the DA content of segments of the ileum and striatum in both groups of animals by the HPLC method with isocratic elution and electrochemical detection (coulometric) [46]. Briefly, ileum sections of circa 0.5 cm or the striatum were cut into small pieces and homogenized in ice-cold perchloric acid (HClO_4_) (0.2 M) by mechanical dissociation using a Potter–Elvehjem polytetrafluoroethylene (PTFE) pestle (Sigma-Aldrich), followed by sonication. The homogenates were subsequently centrifuged (13,000 rpm; 7 min; 4 °C), and the supernatants were filtered (13,000 rpm; 4 min; 4 °C) using 0.2 µm nylon microfilters (Spin-X^®^ Centrifuge Tube Filter, Costar, Corning, Glendale, AZ, USA) and stored at −80 °C until further analysis. The pellets were resuspended in 1 M NaOH and stored at −80 °C for total protein quantification by the bicinchoninic acid (BCA) protein assay (Thermo Fisher Scientific, Waltham, MA, USA). DA was separated in a reversed-phase Grace Platinum EPS C18 column (4.6 mm × 150 mm; 100 Å; 5 µm; W. R. Grace and Co.-Conn, Columbia, MD, USA) with a mobile phase (pH = 4) consisting of 25 mM KH_2_ PO_4_, 2.18 mM 1-heptane sulphonic acid, 50 µM EDTA, and acetonitrile (97:3 *v*/*v*). A Gilson pump (model 307, Fisher Scientific, Porto Salvo, Portugal) maintained a flow rate of 0.3 mL/min. The samples were quantified by an analytical cell (model 5011, ESA Analytical, Dorton Aylesbury, Buckinghamshire, UK) attached to a Coulochem-II electrochemical detector (ESA Analytical). The analytical cell was set at E1 = +250 mV (sensitivity at 100 nA). The DA concentration was determined by comparison with the peak areas of the standards and expressed in nanograms per mg of protein.

### 2.5. Western Blot Analysis

Ileal sections or striata from 6 control-treated and 6 MPTP-treated rats were cut into small pieces and homogenized by mechanical dissociation using a Potter–Elvehjem polytetrafluoroethylene (PTFE) pestle (Sigma-Aldrich) in RIPA lysis buffer [150 mM NaCl, 50 mM Tris (pH 8.0), 5 mM ethylene glycol tetra acetic acid (EGTA), 1% (*v*/*v*) Triton X-100, 0.5% (*m*/*v*) sodium deoxycholate (DOC), and 0.1% (*m*/*v*) sodium dodecyl sulfate (SDS); 4 °C] supplemented with a protease inhibitor cocktail (Roche, Indianapolis, IN, USA). After a 1 h incubation on ice, the homogenates were sonicated and centrifuged (15,500× *g*; 15 min; 4 °C) to pellets of insoluble material. Protein levels were determined by BCA assay, and the supernatants were frozen at −80 °C until analysis. Equal amounts of protein were loaded and separated by sodium dodecyl sulphate polyacrylamide gel electrophoresis (SDS-PAGE) (10%), transferred to polyvinylidene difluoride (PVDF) membranes (Immobilon^®^ Membrane 0.2 µm, Millipore, Madrid, Spain), and blocked with 5% (*m*/*v*) non-fat dry milk in Tris-buffered saline–Tween 20 [TBS-T: 20 mM Tris (pH 7.6), 150 mM NaCl, and 0.1% (*v*/*v*) Tween-20] for 1 h with agitation at room temperature. Subsequently, the membranes were probed using mouse anti-TH (1:1000; Millipore, Burlington, MA, USA), mouse anti-D2R (1:200; Santa Cruz Biotechnology Inc., Dallas, TX, USA), and mouse anti-GFAP (1:1000, Millipore, USA) antibodies overnight at 4 °C. The membranes were then incubated with alkaline phosphatase-conjugated secondary antibodies (1:10,000 anti-mouse, GE Healthcare Bio-Sciences, Piscataway, NJ, USA). Finally, the membranes were visualized with a Fluorescent Image Analyzer FLA 900 Typhoon detector (GE Healthcare Bio-Sciences), using an enhanced chemifluorescence detection reagent (ECF, GE Healthcare Bio-Sciences). To confirm equal protein loading and sample transfer, the membranes were reprobed with mouse anti-β-actin (1:1000; Sigma-Aldrich) or mouse anti-glyceraldehyde 3-phosphate dehydrogenase (GAPDH) (1:1000; Millipore, USA) antibodies. Densitometric analyses were performed using the Image Quant 5.0 software, and the results were normalized against β-actin or GAPDH and then expressed as a percentage of the control group’s protein density.

### 2.6. Immunohistochemistry Analysis

Ileal segments of all 6 control-treated and 6 MPTP-treated rats were fixed in 4% formalin-buffered saline at pH 6.9 and conventionally processed, with subsequent paraffin inclusion. Histological sections (3 µm thick) were deparaffinized in xylene and rehydrated in phosphate buffered saline (PBS) for immunohistochemical studies. Briefly, there was an antigenic retrieval in sodium citrate buffer for 20 min, followed by the appropriate quenching of endogenous peroxidase activity, through a 5-min incubation with 3% diluted hydrogen peroxide. The sections were then incubated with the primary mouse monoclonal antibodies anti-S100β (1:250; Millipore, USA) or anti-D2R (1:50; Santa Cruz Biotechnology Inc.). This was followed by addition of the post-primary [rabbit anti-mouse IgG in 10% (*v*/*v*) animal serum in TBS/0.09%ProClin™ 950; Leica Biosystems Newcastle Ltd., Newcastle, UK] and the polymer linked to peroxidase [Novolink™ Polymer, anti-rabbit Poli-HRP-IgG containing 10% (*v*/*v*) animal serum in TBS/0.09%ProClin™ 950; Leica Biosystems Newcastle Ltd.]. A classical Labeled Streptavidin–Biotin method was used for the immunostaining of GFAP. After antigen retrieval and inhibition of endogenous peroxidase activity, the sections were incubated with normal goat serum (Ultra V block, Labvision, Thermo Fisher Scientific, Fremont, CA, USA) for 5 min at room temperature for the reduction in nonspecific background staining prior to application of the primary antibody anti-GFAP (1:200, Millipore, USA). The sections were further incubated with 1.74% diaminobenzidine (DAB) in a stabilizer solution (*w*/*v*) for 10 min. Finally, the slides were counterstained with 0.02% hematoxylin for 5 min and cover-slipped. Immunostaining analysis was performed by two researchers in a blinded fashion. The rat striatum was used as a positive control for anti-DR2 and anti-GFAP antibodies, and the human colon was used as a positive control for the anti-S100β antibody, according to the manufacturers’ recommendations.

### 2.7. Statistical Analysis

The data are each expressed as the mean ± standard error of the mean (SEM), and the number (*n*) of performed experiments is indicated. The data were analyzed by using an unpaired Student’s *t*-test or one-way ANOVA, followed by Tukey’s multiple comparison test. Significant differences were defined at *p* < 0.05. All analyses were performed using the IBM SPSS Statistics software (version 29.0.0.0., Chicago, IL, USA) and graphs done with GraphPad Prism 8.0.2 (GraphPad Software, San Diego, CA, USA).

## 3. Results

### 3.1. Tonic Contractile Response to DA in Ilea Isolated from Rats Treated with Intranasal MPTP

**Figure 1:**
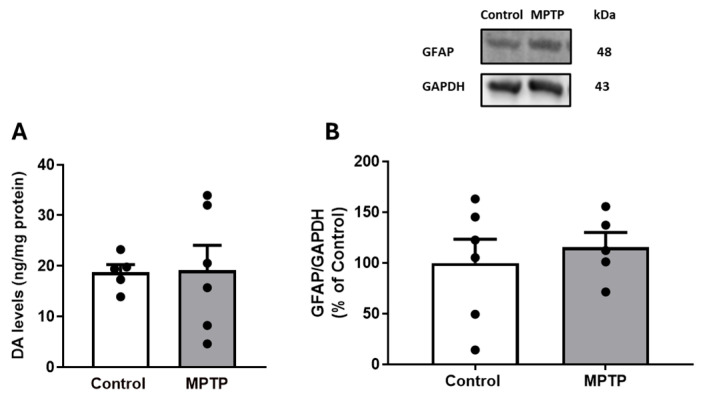
Effect of i.n. MPTP on rat striatal DA total levels by HPLC (**A**) and striatal GFAP density by Western blotting (**B**). Results are expressed as means ± SEMs of 5 to 6 rats; vertical bars represent SEMs. Significance of statistical differences was analyzed by Student’s *t*-test.

**Figure 2:**
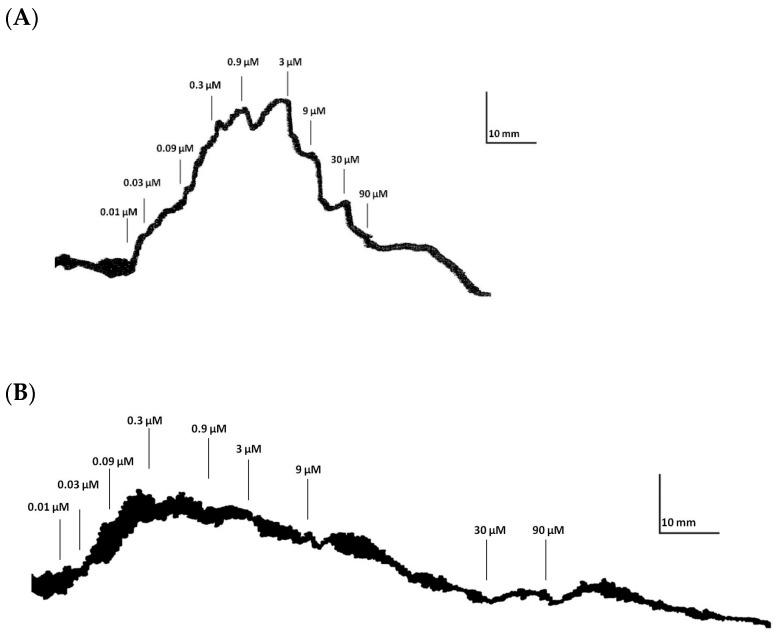
Typical recordings of cumulative concentration–response (CR) curves for dopamine (DA) in ilea isolated from control (**A**) and MPTP-treated (**B**) rats; 10 mm = 3.9 mN; 40 s.

**Figure 3:**
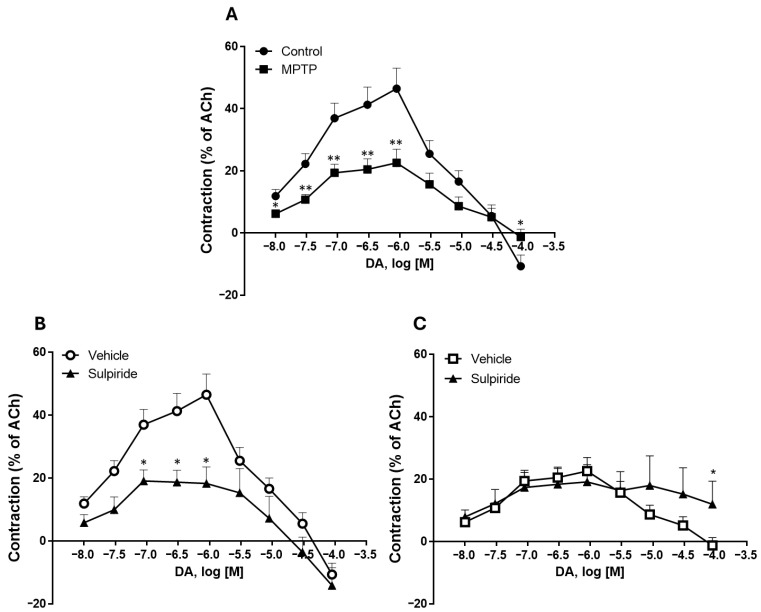
Contractile responses to DA of ilea isolated from control and MPTP-treated rats (**A**). Contractile responses to DA of ilea from Control (**B**) and MPTP-treated (**C**) rats in the absence (vehicle) or in the presence of 10 µM of sulpiride. Results are expressed as means ± SEMs; vertical bars represent SEMs. Significance of statistical differences was analyzed by Student’s *t*-test. * *p* < 0.05; ** *p* < 0.01 vs. Control/Vehicle.

Herein, we tested the tonic contractile response to DA in ilea from MPTP-treated rats. Firstly, one should acknowledge that MPTP treatment did not alter body weight compared with control rats (Supplementary Figure S1). Secondly, we confirmed that this MPTP dose did not statistically change the striatal DA content (MPTP: 19.2 ± 4.9 ng/mg of protein vs. control: 18.7 ± 1.5 ng/mg of protein (*n* = 5–6); *p* > 0.05) or striatal GFAP levels (MPTP: 115.5 ± 14.6% vs. control: 100.0 ± 23.4% (*n* = 5–6); *p* > 0.05) (Figure 1A,B). This suggests that this MPTP dosing regimen was not inducing striatal DAergic neurotoxicity at this time-point. We then ascertained that MPTP treatment did not significantly change the ileum smooth muscle contractile response to 100 μM of exogenous ACh, since we expressed the results as percentages of ACh-induced maximum contraction (MPTP: 29.64 ± 3.45 mN (*n*= 16) vs. control: 21.55 ± 2.16 mN (*n* = 16); *p* > 0.05; Supplementary Figure S2). As shown by the representative recording of cumulative CR curves for DA in the ileum of control and MPTP-treated rats (Figure 2A,B), cumulative concentrations of DA (0.01–90 µM) induced contractions in the ileum at lower concentrations (0.01–0.9 µM), followed by relaxation responses at higher concentrations (3–90 µM), accompanied by a reduction in baseline tone (Figure 3A). The contractile response of the ilea from control-treated rats to DA was not statistically altered by atropine, a non-selective muscarinic receptor antagonist (see Figure S3, Supplementary Data). In addition, MPTP imposed a statistically significant decrease in the DA maximum contractile effect and attenuated the relaxation responses seen at the highest DA concentrations in control animals (Figure 3A). Furthermore, pre-incubation of ileum segments with 10 µM of sulpiride, a selective D2R antagonist, significantly reduced the DA maximum contraction in the control group (Figure 3B), without modifying the relaxation response triggered by higher DA concentrations. Meanwhile, sulpiride abrogated the relaxation response seen in MPTP-isolated ilea, without altering the profile seen for the lower DA concentrations (Figure 3C). In Table 1, it is possible to see the effect of DR2 blocking by sulpiride on the maximum contractile response to DA in the ilea from control and MPTP rats.

### 3.2. Dopaminergic and Gliosis Markers in Ilea of Rats Treated with Intranasal MPTP

**Figure 4:**
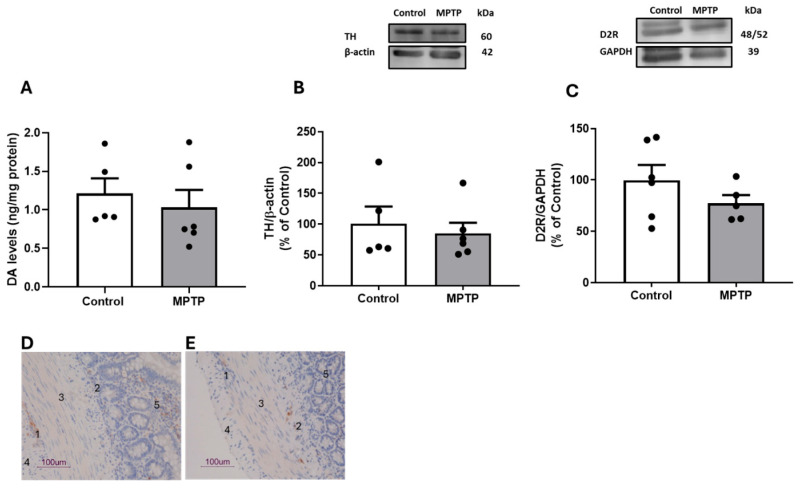
Effect of i.n. MPTP on rat ileum DA homeostasis: (**A**) DA total levels by HPLC; TH (**B**) and D2R (**C**) densities by Western blotting; representative D2R immunohistochemical findings in the isolated ilea of control (**D**) and MPTP-treated (**E**) rats. Results are expressed as means ± SEMs of 5 to 6 rats; vertical bars represent SEMs. Significance of statistical differences was analyzed by Student’s *t*-test. *p* < 0.05 was considered significant. D2R immunoreactivity appears in brown, counterstained with hematoxylin, and is more intense in ganglion cells of myenteric plexus (1) compared with submucosal plexus (2) in ilea from both control and MPTP groups. Smooth muscle cells of circular (3) and longitudinal layers (4) are devoid of D2R immunoreactivity. Note that D2R labeling can also be seen in mucosal tissues (5) in both control and MPTP ilea. Scale bars: 100 µm.

The effects of MPTP administration on DAergic markers in the rat ileum, including DA total content, TH, and D2R density, are shown in Figure 4A–C. MPTP did not induce significant changes in DA tissue levels (MPTP: 1.0 ± 0.2 ng/mg of protein vs. Control: 1.2 ± 0.2 ng/mg of protein; *p* > 0.05), TH (MPTP: 84.9 ± 17.4% vs. Control: 100.0 ± 28.1%; *p* > 0.05), and D2R densities (MPTP: 77.5 ± 7.8% vs. Control: 100.0 ± 18.4%; *p* > 0.05) compared with the control group. D2R was further analyzed by immunohistochemistry to unveil its anatomical location (Figure 4D,E).

Indeed, DR2 immunostaining was found in the nervous plexuses, and it was more intense in the myenteric plexus than in the submucosal plexus of the ilea from both control and MPTP groups, but not in the smooth muscle cells of both circular and longitudinal muscles.

**Figure 5:**
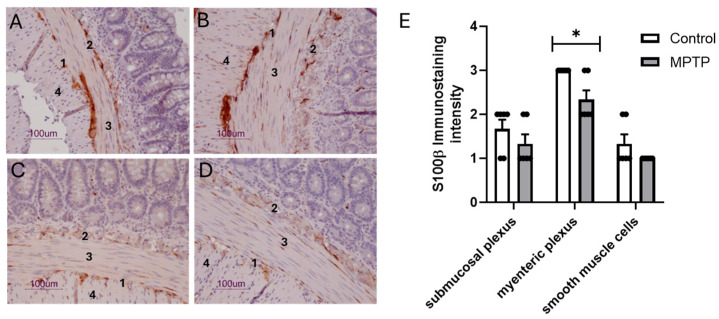
Representative S100β immunohistochemical findings in isolated ilea from control (**A**,**B**) and MPTP-treated (**C**,**D**) rats. S100β immunoreactivity appears in brown, counterstained with hematoxylin. S100β-positive cells in ganglion cells of both myenteric (1) and submucosal (2) plexuses and smooth muscle cells of circular (3) and longitudinal layers (4). Scale bars: 100 µm. (**E**) The rating for intensity and extension of S100β immunostaining was set, ranging from 0 (no staining) to 3 (intense and extensive staining), respecting tissue specificity scoring when adequate. MPTP-treated group evidenced a significant decrease in S100β immunoreactivity in myenteric plexus when compared with control group, whereas submucosal plexus and smooth muscle cells showed a similar S100β labeling pattern compared to control. Data are expressed as means ± SEMs of 6 rats/group. Significance of statistical differences was analyzed by Student’s *t*-test. * *p* < 0.05 vs. Control.

Figure 5 shows representative images displaying S100β immunoreactivity in the ilea of control (Figure 5A,B) and MPTP (Figure 5C,D) rats. S100β immunostaining was widespread throughout all layers of the intestinal wall, from the mucosa to the longitudinal muscle. However, according to the rating for intensity and extension, there was a significant decrease in the S100β immunoreactivity in the myenteric plexus of the MPTP-treated group compared with the control group (*p* < 0.05; Figure 5E), whereas submucosal plexus and smooth muscle cells showed a similar S100β labeling pattern between both groups.

**Figure 6:**
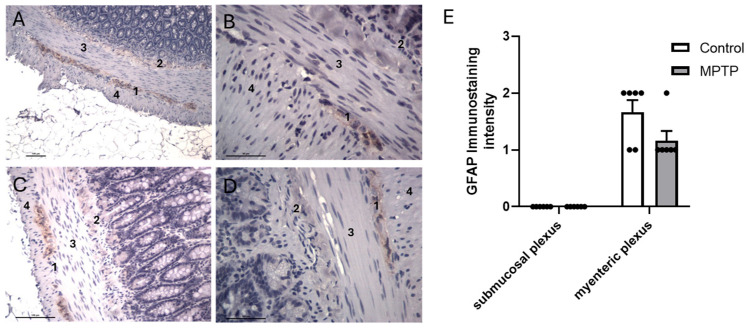
Representative GFAP immunohistochemical findings in ilea isolated from control (**A**,**B**) and MPTP-treated (**C**,**D**) rats. GFAP immunoreactivity appears in brown, counterstained with hematoxylin. GFAP-positive cells in myenteric (1) plexus but not in submucosal (2) plexus and smooth muscle cells of circular (3) and longitudinal layers (4). Scale bars: 100 µm. (**E**) The rating for intensity and extension of GFAP immunostaining was set, ranging from 0 (no staining) to 3 (intense and extensive staining), respecting tissue specificity scoring when adequate. MPTP-treated group showed similar GFAP pattern compared to control. Data are expressed as means ± SEMs of 6 rats/group. Significance of statistical differences was analyzed by Student’s *t*-test. *p* < 0.05 was considered significant.

According to representative images, GFAP-positive cells were found in the myenteric plexus but not in the submucosal plexus or smooth muscle layer cells in the ilea from control (Figure 6A,B) and MPTP-treated rats (Figure 6C,D). GFAP semi-quantification showed that there was a decrease in GFAP immunostaining in MPTP-treated rats compared with the control group, which did not reach significance (*p* > 0.05; Figure 6E).

## 4. Discussion

Gastrointestinal dysfunction is the most common non-motor symptom of PD [47]. GI impairments are associated with the alteration of DAergic neurons in the myenteric plexus [48]. However, a full understanding of the pathophysiology of GI symptoms in PD is still lacking. Interestingly, Choi et al. [49] reported an increase in iNOS expression in ileal tissues from MPTP-treated mice since the asymptomatic stages of the disease. Nevertheless, contractile changes in the small intestine are poorly studied in animal models of PD.

Herein, we used an experimental model consisting of a single i.n. administration of MPTP to rats that we and others have been employing to investigate early pre-motor PD symptoms, such as olfactory and memory impairments, depressive-like behaviors, reduced levels of brain neurotransmitters (dopamine, serotonin, and noradrenaline) and their markers, as well as increased oxidative stress and neuroinflammation [38,39,50,51,52,53]. This PD model is aligned with the argument that the olfactory and gastrointestinal systems are gateways to environmental factors that might have an important role in triggering and/or propagating the pathology of PD [35]. Additionally, it was demonstrated that MPTP is also a DA neurotoxin in the ENS [47,54]. Moreover, evidence suggests that the neurodegenerative process that leads to PD starts in the ENS and spreads via the vagus nerve to the brain [35,55]. We are now analyzing, pharmacologically, the effects of DA on rat ileum contractility 12 days post-MPTP administration, a time-point where there are no overt motor impairments [50,53]. This is consistent with the absence of striatal DAergic neurotoxicity and astrogliosis seen herein. Additionally, rats seem to display normal nutritional status, as gauged by their body weight.

DA is released by enteric neurons and modulates the motility of the small intestine smooth muscle cells [56]. Before testing DA, segments of the ileum were challenged with ACh to induce maximum contraction of the ileum smooth muscle. In fact, ACh is a major excitatory neurotransmitter in the ENS, which explains the importance of cholinergic mechanisms in the regulation of intestinal motility [57]. Given that ileum smooth muscle maximal contraction was not significantly different between control and MPTP rats, this suggests that MPTP does not impair cholinergic control of ileal contractility.

We showed that DA elicited a concentration-dependent contractile response in the ilea of rats (0.01–0.9 µM DA), which was followed by a concentration-dependent relaxation response when using doses from 3 up to 90 µM DA. The contractile effect of DA was moderate, considering that Emax was circa 50% of the ACh-induced contraction. While in different sections of the small intestine, Kirschstein et al. [56] also reported apparently opposite responses to DA, i.e., DA induced contraction in the isolated proximal small intestine but relaxation in the distal small intestine of rats, although different technical approaches were used between the labs. While we used a lower DA concentration range (0.01–90 µM) and obtained cumulative CR curves from a stable baseline with little or no phasic contractions, Kirschstein et al. [56] used the highest DA concentration range (0.1–1000 µM) and obtained curves with independent additions from a stable baseline tone with regular phasic contractions. Nonetheless, these authors showed that DA induced a contractile response in duodenum strips from rats, followed by relaxation in the ileum. These authors further showed that both the D1-like receptor antagonist SCH23390 and the D2-like receptor antagonist raclopride blocked the DA-induced contraction in the duodenum. However, the relaxations in the ileum were not influenced by DA receptor antagonists. Importantly, we herein demonstrated that D2R mediated DA-induced contractions in the ilea of control rats, since sulpiride significantly attenuated DA-induced ileum contraction. Little is known about the characterization of D2R in the rat GI tract and its role in intestinal motility. We not only characterized D2R pharmacologically and neurochemically but also studied its localization in rat ilea. Immunohistochemical analysis showed that D2R immunoreactivity was more intense in ganglion cells of the myenteric plexus than in the submucosal plexus in control animals. Meanwhile, smooth muscle cells did not show D2R immunoreactivity. Our data are consistent with transcripts encoding D2R having been detected in myenteric neurons of the mouse ileum [18]. The ENS is a semiautonomous network of nerves that includes a superficial myenteric plexus and a deeper submucosal plexus [58] and is responsible for local control of gut motility and function. The myenteric plexus lies between longitudinal and circular smooth muscle layers and mainly regulates muscle motility, and the submucosal plexus is embedded in the submucosal connective tissue and mainly regulates mucosal functions, including secretory activity. Therefore, D2R localization, which is mainly in the myenteric plexus, is consistent with its role in gut contractility [59]. Considering that the DA-induced contractile effect was not significantly modified by atropine and that D2Rs are expressed in the myenteric plexus, one can argue that the DA effect is mediated by presynaptic D2-like receptors. It is tempting to speculate that DA may inhibit the inhibitory motor neurons in the ileum, thus releasing the tonic relaxation imposed by mainly nitric oxide (NO), and also by vasoactive intestinal peptide (VIP) and adenosine triphosphate (ATP), which are used by these neurons [60]. In fact, the role of enteric inhibitory neurons in intestinal motility is being increasingly recognized [61]. Colucci et al. [62] demonstrated that altered colon propulsive activity was attributable to the decreased expression of D2R in both the proximal and distal colon and associated with changes in the expression of enteric inhibitory neurotransmitters, VIP and NO, in the myenteric plexus. The disinhibition of inhibitory neurons by DA in our model needs to be demonstrated in future work. Our results provide the first evidence that i.n. MPTP administration attenuates DA contractile response. Indeed, the DA-induced contraction was only 22% of that induced by ACh in ilea from MPTP rats, and sulpiride further blocked the relaxation effects of DA. In fact, it is interesting to see that this relaxation was mediated by D2R in MPTP rats and that the contraction was mediated by D2R in control rats. Moreover, MPTP did not change D2R immunolabeling and D2R density measured by immunohistochemistry and Western blot, respectively. Overall, this suggests that D2R signaling contributed to contractile and relaxation responses in the rat ilea in the control and MPTP groups, respectively. This warrants further investigation in the future. It is also tempting to speculate that other receptors, including D1R, may also be involved in ileum contractility. As mentioned before, Kirschstein et al. [56] demonstrated that DA induces contraction in the proximal isolated small intestine of rats by activating both D1 and D2 receptors.

The enteric dysfunction reported herein is aligned with Natale et al.’s [63] and Colucci et al.’s [62] observations in PD experimental models using MPTP and 6-OHDA, respectively. However, these authors focused on colonic function, whereas we addressed ileum motility. Moreover, Natale et al. [63] reported, along with the delay in colon motility, a loss of TH-positive cells in the gut 10 days after MPTP administration (20 mg/kg MPTP; three i.p. injections). Additionally, there is evidence to suggest that the D2R-containing striatal medium spiny neurons of the indirect pathway may be preferentially affected after DA depletion in PD experimental models [64]. Therefore, it was mandatory to evaluate if DAergic neurotoxicity was in place in the ilea from our MPTP-exposed rats. Importantly, MPTP did not significantly change tissue levels of DA and TH (the rate-limiting enzyme in catecholamine biosynthesis) in comparison with controls, which means that in our rat MPTP model, DA-induced ileal contractility alterations occur with preserved ileal and striatal DA homeostasis. Both DA and TH are likely markers of intrinsic DAergic neurons residing in the enteric plexus, as suggested by Li et al. [65].

On the contrary, PD patients have been reported to present a lower level of DA in the muscularis externa of the colon [20]. In pre-clinical studies, TH-positive neurons in the injured substantia nigra of the brain of MPTP-injected cynomolgus monkeys showed up to an 83.95% reduction [66]. Moreover, histopathological examination showed marked damage to both enteric nerves and TH neurons, along with significant disruption of mucosal structure, intestinal barrier integrity, and motility in PD monkeys across all four intestinal segments, including the duodenum, ileum, transverse colon, and rectum. Also, there was a reduction in DA neurons in the mouse ileum 10 days after MPTP administration (15 mg/kg MPTP; four i.p. injections) [47]. Another study found that two subacute administrations of MPTP induced DAergic neuron injury and inflammation in the midbrain and ileum in mice, and impaired intestinal barrier function and gut microbiota disorders closely related to administration [67]. It is worth mentioning that species, routes of MPTP administration (i.n. vs. i.p.), dosing (single vs. multiple MPTP administration), and differences between analyzed tissues (ileum vs. colon) could account for the lack of DAergic neurotoxicity seen in our model, as opposed to other studies. Additionally, one should stress that at our time-point (12 days after i.n. MPTP), overt neurodegeneration is not yet in place.

PD pathology is also associated with chronic neuroinflammation, controlled mainly by resident glial cells, including astrocytes [68]. These glial cells express and release S100β (a calcium-binding protein), which promotes neuronal survival and proliferation of astrocytes at nanomolar concentrations [69]. However, S100β at micromolar concentrations acts as a cytokine or damage-associated molecular pattern protein, playing a major role in neurodegenerative diseases associated with dysregulated glial cell proliferation and neuroinflammation. Additionally, GFAP is known to reflect astrogliosis in alignment with S100β [70]. Enteric glial cells, which are similar to CNS astrocytes, participate in the homeostasis of the GI tract. One should consider that enteric glial cells are likely to play a role in MPTP gut toxicity since they may engage in metabolizing MPTP (proneurotoxin) into MPP+ (active metabolite), which is then uptaken mostly by the dopamine transporter (DAT) in intrinsic dopamine neurons in the ENS, causing mitochondrial complex I inhibition, as previously described for astrocytes in the CNS [71].

The correlation between overexpression and release of S100β by enteroglial cells with intestinal inflammatory conditions has already been shown [69]. Growing evidence suggests the implication of the S100β protein in the pathogenesis of PD [72]. However, S100β has been overlooked in ENS pathology triggered by MPTP. To date, GFAP and S100β remain the most commonly used markers to identify enteric glia [73].

Herein, we provide the first evidence that S100β immunoreactivity was decreased in the myenteric plexus but not in the submucosal plexus in the MPTP group when compared with the control group. Moreover, MPTP showed a tendency to decrease GFAP immunoreactivity in the myenteric plexus. Additionally, this glial marker is not expressed in the submucosal plexus in our model. Importantly, it was shown that mammalian enteric glia are heterogeneous and that GFAP expression is limited to a subset [73,74]. More than half of the mucosal and submucosal glia in the ileum and colon do not express GFAP [73]. In contrast, S100β is expressed by virtually all enteric glia. Human data showed that GFAP, but not S100β, was elevated in the ascending colon of PD patients [31]. Colonic biopsies from asymptomatic PD patients showed activation of enteric glial cells and an increase in S100β-positive cells [75]. Finally, duodenal biopsies from patients with advanced PD and untreated patients with early PD showed increased size and density of GFAP-positive enteroglial cells, suggesting reactive gliosis [76,77]. On the one hand, mice receiving four i.p. MPTP (8 mg/kg) injections at 2 h intervals did not show alterations in the relative expression of myenteric GFAP in the ilea, 5 days post-MPTP injection [78]. Nonetheless, DAergic neurons were reduced by approximately 60%, and pro-inflammatory macrophages were increased by approximately 50% in the myenteric plexus of MPTP mice compared with intact controls. On the other hand, after administering chronic MPTP/probenecid injections to mice, the GFAP expression levels were dramatically decreased, while the TH expression levels in the stomach were significantly upregulated. These alterations were concomitant with an increase in abnormal aggregated and nitrated α-synuclein in the TH-positive neurons and enteric glial cells of the gastric myenteric plexus. Overall, MPTP models and clinical data show a wide range of alterations in S100β and GFAP that can illustrate a diverse array of experimental models and biopsies analyzed. Nonetheless, the apparent decrease in S100β and GFAP in the myenteric plexus seen herein may suggest an impaired enteroglial function that accompanies the disturbed ileal motility in our model.

In summary, we provide pioneering functional, neurochemical, and morphological evidence showing that this experimental MPTP model has DA-dependent ileum motile dysfunction accompanied by enteroglial changes within the myenteric plexus, but with preserved DA markers. Further investigation is warranted to further understand the involvement of D2R and possibly D1R in the ileal contractile response of DA. It is also relevant to establish a correlation between glial alterations and ileal contractility in MPTP models. This study further proposes that autonomic GI dysfunction has potential sensitivity as a clinical biomarker of the premotor PD phase.

## Figures and Tables

**Table 1:** E_max_ values for DA in ilea isolated from Control and MPTP-treated rats.

	Control	Control + Sulpiride	MPTP	MPTP + Sulpiride
E_max_	46.44 ± 6.57	18.25 ± 5.23 *	22.55 ± 4.35 *	19.05 ± 5.25 *

E_max_ = maximum contraction in % of ACh-induced contraction. Data are expressed as means ± SEMs of 5–13 experiments. Statistical differences were evaluated by ANOVA followed by Tukey’s multiple comparisons test. * *p* < 0.01 vs. Control.

## Data Availability

Data presented in this study is contained within the article and Supplementary Materials. Further inquiries can be directed to the corresponding author.

## Supplementary Materials

The following supporting information can be downloaded at https://www.mdpi.com/article/10.3390/biomedicines13102400/s1. Figure S1: MPTP treatment effects on Wistar rat’s body weight; Figure S2: Contractile responses to 100 µM ACh of ilea from both Control and MPTP-treated rats; Figure S3: Contractile response of the Wistar rat’s ileum to DA, in the absence or in the presence of 1µM Atropine.
