# Supplementary material for: Intranasal 1-Methyl-4-phenyl-1,2,3,6-tetrahydropyridine (MPTP) Administration Hampered Contractile Response of Dopamine in Isolated Rat Ileum
Source: Biomedicines. 2025 Sep 30;13(10):2400. doi: 10.3390/biomedicines13102400 (PMC12562183; doi:10.3390/biomedicines13102400)
Supplement: Supplementary file 1 [file biomedicines-13-02400-s001.zip › biomedicines-3847951-supplementary.pdf]

Supplementary data

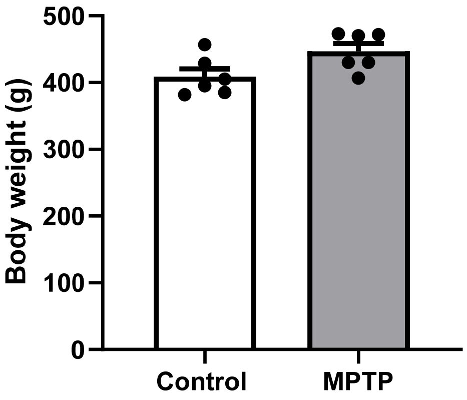

Figure S1: MPTP treatment effects on Wistar rat's body weight. The results are expressed as mean ± S.E.M. of 6 animals/group; vertical bars represent S.E.M. The significance of the statistical differences was analyzed by Student's t-test. $p < 0.05$ was considered significant.

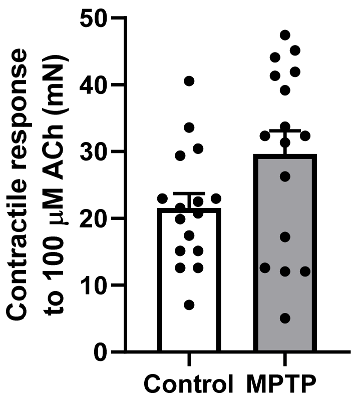

Figure S2: Contractile responses to 100 µM ACh of ilea from both Control and MPTP-treated rats. The results are expressed as mean ± S.E.M. of 16 strips from 6 animals/group; vertical bars represent S.E.M. The significance of the statistical differences was analyzed by Student's t-test. $p < 0.05$ was considered significant.

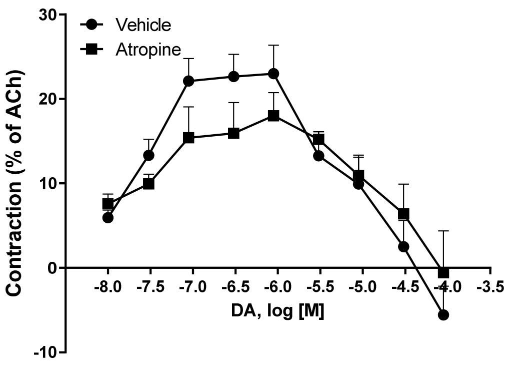

Figure S3: Contractile response of the Wistar rat's ileum to DA, in the absence or in the presence of 1µM Atropine. Data corresponds to the mean ± S.E.M. of 20 to 6 strips from 4 animals; vertical bars represent S.E.M. The significance of the statistical differences was analyzed by Student's t-test. $p < 0.05$ was considered significant.
